# Cytoskeleton Rearrangement in Podocytopathies: An Update

**DOI:** 10.3390/ijms25010647

**Published:** 2024-01-04

**Authors:** Sijia Ma, Yang Qiu, Chun Zhang

**Affiliations:** Department of Nephrology, Union Hospital, Tongji Medical College, Huazhong University of Science and Technology, Wuhan 430022, China; masijia1018@hust.edu.cn (S.M.); dryangqiu@hust.edu.cn (Y.Q.)

**Keywords:** cytoskeleton rearrangement, podocytopathies, podocyte injury, actin

## Abstract

Podocyte injury can disrupt the glomerular filtration barrier (GFB), leading to podocytopathies that emphasize podocytes as the glomerulus’s key organizer. The coordinated cytoskeleton is essential for supporting the elegant structure and complete functions of podocytes. Therefore, cytoskeleton rearrangement is closely related to the pathogenesis of podocytopathies. In podocytopathies, the rearrangement of the cytoskeleton refers to significant alterations in a string of slit diaphragm (SD) and focal adhesion proteins such as the signaling node nephrin, calcium influx via transient receptor potential channel 6 (TRPC6), and regulation of the Rho family, eventually leading to the disorganization of the original cytoskeletal architecture. Thus, it is imperative to focus on these proteins and signaling pathways to probe the cytoskeleton rearrangement in podocytopathies. In this review, we describe podocytopathies and the podocyte cytoskeleton, then discuss the molecular mechanisms involved in cytoskeleton rearrangement in podocytopathies and summarize the effects of currently existing drugs on regulating the podocyte cytoskeleton.

## 1. Introduction

Podocytopathies are kidney diseases caused by podocyte injury, characterized by proteinuria. Despite the lack of precise epidemiological data, it is evident that the prevalence of podocytopathies is increasing globally, and they are the leading cause of end-stage renal disease (ESRD). However, even with the high prevalence and various complications, the current treatment for podocytopathies is mainly symptomatic and poorly effective, such as immunosuppressive drugs, renin–angiotensin system antagonists, and diuretics. Moreover, the disease often relapses after treatment with these drugs [1].

Podocytes are terminally differentiated cells that, together with the glomerular basement membrane (GBM) and endothelial cells, constitute the glomerular filtration barrier (GFB) [2]. Podocytes comprise the cell body, primary processes, and secondary foot processes (FPs), forming an octopus-like structure [3]. The interdigitating FPs of adjacent podocytes build a special connection, namely the slit diaphragm (SD) [4]. As the critical component of the kidney filtration units, podocytes maintain their unique cellular structure through an intricate and coordinated network of cytoskeletons [5]. Direct or indirect podocyte injury perturbs the cytoskeleton, especially the actin filaments (AFs), resulting in foot process effacement (FPE), proteinuria, and ultimately chronic kidney disease (CKD). Podocyte injury poses a threat to the glomerulus and is the determinant of podocytopathies, such as focal segmental glomerulosclerosis (FSGS), minimal change disease (MCD), membranous nephropathy (MN), and diabetic kidney disease (DKD) [6].

Actin cytoskeleton dysfunction is the principal mechanism of FPE in podocytes [7]. Podocytes are extremely motile, particularly after injury, and the morphology of podocytes is highly dependent on the actin cytoskeleton, which is regulated by cytoskeleton-associated proteins [8]. To adapt to various stresses, such as genetic mutations and significant mechanical forces, podocytes rearrange the cytoskeleton to maintain its distinct architecture and intact filtration characteristic [9]. A series of SD proteins are regulated and altered to participate in podocyte cytoskeleton rearrangement [9,10,11]. These studies are of great significance for elucidating the mechanism of podocyte cytoskeleton rearrangement in podocytopathies. Accordingly, targeting the podocyte cytoskeleton holds significant therapeutic promise.

In this review, we discuss recent findings shedding new insight into the cytoskeleton rearrangement in podocytopathies, summarize the effects of currently available drugs on the cytoskeleton, and explore more potential therapeutic approaches.

## 2. Overview of Podocytopathies

Podocytopathies are glomerular disorders characterized by podocyte injury with proteinuria and can ultimately progress to CKD [1]. The primary forms of podocytopathies usually refer to FSGS and MCD [12]. Podocytopathies can also be secondary to systemic diseases of a broad array of etiologies, including genetic variants and environmental triggers such as immune, metabolic, infectious, toxic, and hemodynamic factors [13]. The immune system plays a pivotal role in podocyte injury, for example, in MN and lupus nephritis (LN). Long-term, poorly controlled diabetes mellitus leads to DKD accompanied by hemodynamic changes, resulting in glomerular hyperfiltration [1]. The concept of podocytopathies localizes the damage to podocytes. FPE is the primary morphological feature in podocytopathies. Persistent and severe injury induces the detachment of podocytes from the GBM, leading to the loss of podocytes and consequently glomerulosclerosis. These changes are tightly associated with excessive proteinuria [1,14].

## 3. Composition and Structure of the Podocyte Cytoskeleton

**Figure 1:**
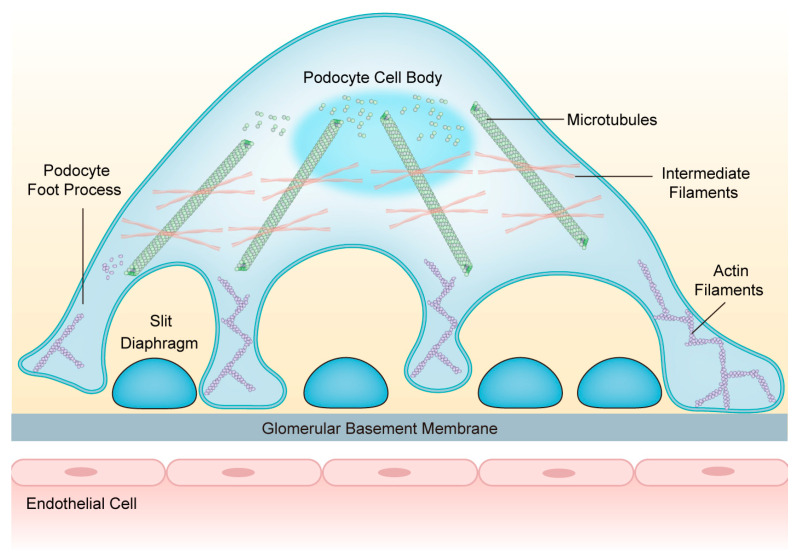
The structure of podocytes and the network of podocyte cytoskeletons. Podocytes consist of cell bodies, primary processes, and secondary foot processes; adjacent foot processes form the slit diaphragm. The cell bodies and primary processes are based on microtubules and intermediate filaments, while the foot processes mainly depend on the actin filaments. Abbreviations: FPs: foot processes; SD: slit diaphragm; GBM: glomerular basement membrane; MTs: microtubules; IFs: intermediate filaments; AFs: actin filaments.

Podocytes are equipped with a well-orchestrated cytoskeletal backbone consisting of the actin cytoskeleton, microtubules (MTs), and intermediate filaments (IFs). It is indispensable for maintaining GFB integrity and regulating cell structure, stability, motility, cell adhesion, and SD insertion [15]. The cell body and major processes of podocytes are based on MTs and IFs, while the FPs mainly rely on the actin cytoskeleton [16] (Figure 1). Under physiological conditions, the podocyte cytoskeleton is in dynamic equilibrium to preserve highly organized cellular function [17].

### 3.1. Actin Cytoskeleton

The actin cytoskeleton is the core component of podocytes and is mainly abundant in the FPs. It plays a dominant role in establishing and maintaining the distinct architecture of podocytes [7]. The composition of the actin cytoskeleton in the FPs contains two groups: central actin bundles above the SD and a network of cortical actin fibers beneath the curved plasma membrane of the FPs [18]. The AFs are composed of globular actin monomers and are in a highly dynamic state, constantly polymerizing and depolymerizing, mediated by the interplay between multiple actin-associated proteins [19]. Many biochemical factors contribute to the regulation of AFs assembly and disassembly, such as the actin-related proteins 2/3 (Arp2/3) complex, cofilin, and transgelin. In addition, mechanical forces can also influence the assembly and disassembly of AFs [10]. The actin cytoskeleton is strongly sensitive and responsive to the mechanical stress that podocytes face [20]. The AFs provide a structural and functional basis, thus building the cytoarchitecture of podocytes and enhancing adhesion to the extracellular matrix [7,16]. The central actin bundles are engaged in generating tensile forces, whereas the cortical actin network is necessary to uphold the elaborate morphology of FPs [15].

### 3.2. Microtubules

Podocytes contain a sophisticated and ordered microtubule network, one of the principal cytoskeletal elements within the cell body, and the primary processes of podocytes [21]. MTs are highly dynamic polymers that assemble from α- and β-tubulin heterodimers with an uneven polarity, which refers to fast-growing (plus) ends and slow-growing (minus) poles in coexistence [22]. MTs actively participate in the maintenance of cellular architecture and intracellular transport of proteins and organelles within podocytes, particularly playing a pivotal role in the morphogenesis of podocytes and process growth, branching, and elongation [23]. Podocyte process formation and bipolar orientation mechanically depend on the correct assembly of MTs [24]. The dynamic balance of MT assembly and disassembly is closely regulated by microtubule-associated proteins (MAPs) such as MAP3 and MAP4 [25].

### 3.3. Intermediate Filaments

IFs are composed of heterogeneous proteins with an intermediate size [26]. Three IF proteins, including vimentin, nestin, and desmin, are abundantly expressed in the cell body and primary processes of podocytes [27]. The first established role of IFs is to provide mechanical support for podocytes, thus stabilizing the cellular biomechanics [28]. IFs significantly modulate the elasticity of the glomerulus and communicate constantly with actin [29]. In addition, IFs impact the organization of MTs and microfilaments and the control of cell cycle, cell survival, and apoptosis [26]. As a sensitive marker of podocyte injury, desmin is upregulated in response to mechanical stresses [27]. Nestin contributes to the metanephric mesenchyme-derived structures of the kidney [30].

## 4. Cytoskeleton Rearrangement in Podocytopathies and Podocyte-Centric Diseases

### 4.1. Focal Segmental Glomerulosclerosis

FSGS is a histological pattern of diverse clinical–pathologic syndromes characterized by focal and segmental glomerular scarring, podocyte injury, and proteinuria [31]. Studies have provided clues that podocyte cytoskeleton rearrangement is involved in the pathogenesis of FSGS [32]. During the development of FSGS, the expression or distribution of cardinal SD proteins altered, and the podocyte cytoskeleton was rearranged [33].

**Figure 2:**
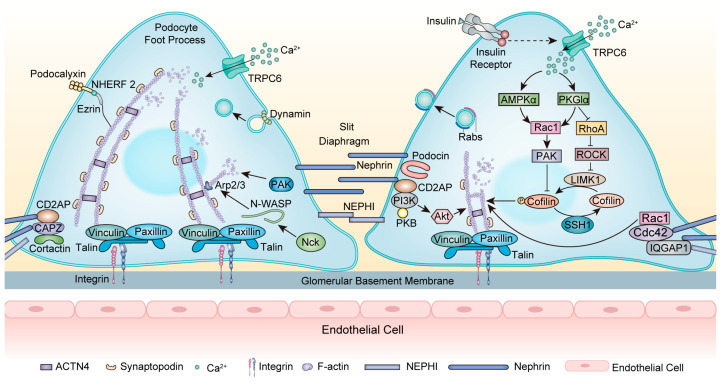
Modulation of the actin cytoskeleton within the foot processes. At the SD, nephrin acts as a signaling hub to interact with other proteins. Nephrin interacts with CD2AP with a subunit of PI3K and then activates the Akt kinase pathway, leading to actin cytoskeleton rearrangement. The adaptor protein Nck binds to phosphorylated nephrin and N-WASP, stimulating the Arp2/3 complex, which triggers actin polymerization. The scaffolding protein IQGAP1 interacts with nephrin and regulates actin dynamin. In DKD, insulin modulates actin cytoskeleton rearrangement by activating TRPC6, affecting AMPKα and PKGIα, and then regulating RhoA and Rac1. These interactions can regulate the PAK/cofilin-dependent signaling pathway. Dynamin functions in endocytosis and regulates the scission and release of vesicles. Rabs are localized in close proximity to vesicles within FPs and serve as predominant mediators of vesicle trafficking while closely interacting with cytoskeleton proteins. Abbreviations: FPs: foot processes; SD: slit diaphragm; GBM: glomerular basement membrane; AFs: actin filaments; TRPC6: Transient receptor potential channel 6; CD2AP: CD2-associated protein; CAPZ: F-actin capping protein; PAK: p21-activated kinases; Nck: Non-catalytic region of tyrosine kinase; N-WASP: neuronal Wiskott–Aldrich syndrome protein; Arp2/3: actin-related proteins 2/3; ACTN4: α-actinin-4; NEPH1: nephrin-like protein 1; NHEFR 2: Na^+^/H^+^ exchange regulatory cofactor 2; IR: insulin receptor; AMPKα: AMP-activated protein kinase α; PKGIα: protein kinase G type Iα; ROCK: Rho-associated protein kinase; IQGAP1: IQ domain GTPase-activating protein 1; PI3K: phosphoinositide 3-kinase; PKB: protein kinase B; Ca^2+^: calcium; F-actin: filamentous actin.

As a transmembrane protein, nephrin forms a zipper-like structure in the SD and acts as a signaling hub [34] (Figure 2). Nephrin (encoded by *NPHS1*) is essential for maintaining cytoskeleton stability. *NPHS1* mutations typically cause congenital nephrotic syndrome (NS) of the Finnish type and also FSGS in adults [35,36]. Nephrin directly interacts with podocin (encoded by *NPHS2*) to transport nephrin to the plasma membrane [37]. *NPHS2* mutations lead to the misfolding and mislocalization of podocin and interrupt the proper trafficking of nephrin, causing FSGS [38]. In addition, nephrin interacts with CD2AP with a subunit of PI3K and subsequently activates the Akt kinase pathway, which is necessary for regulating the actin cytoskeleton [39]. CD2AP mutations are related to primary FSGS, and mutant CD2AP displays a significantly decreased F-actin binding efficiency [40]. Moreover, the actin crosslinking protein ACTN4 interacts with nephrin [41]. Mutations in ACTN4 caused irreversible reductions in the actin cytoskeleton as well as stiffer actin dynamics, causing an autosomal dominant FSGS [42]. After nephrin phosphorylation, the adaptor protein Nck binds to phosphorylated nephrin and neuronal Wiskott–Aldrich syndrome protein (N-WASP), then stimulates the Arp2/3 complex, which triggers actin polymerization and accelerates AFs assembly [43]. Nck1 and Nck2 cooperate to control podocyte adhesion and actin cytoskeleton rearrangement [44]. Podocyte-specific deletion of Nck1/2 in mice results in the FSGS phenotype [45].

The coordination of RhoA and Rac1/Cdc42 activity is vital to maintaining the actin cytoskeleton. Increased activity in RhoA and Rac1 and decreased activity in Cdc42 induce actin cytoskeleton disruption, FPE, and FSGS [46]. Synaptopodin can regulate RhoA and Rac1, therefore modulating actin dynamics [47]. Loss of synaptopodin decreases RhoA activation and increases Rac1 activation, thus altering the actin cytoskeleton [48]. Li and colleagues recently identified an unconventional myosin, Myo9A, as a novel podocyte cytoskeletal component that regulates RhoA activity. Myo9A^R701X/D^ mutant mice developed FSGS and showed increased RhoA activity and decreased Myo9A–actin—calmodulin interaction in podocytes. Therefore, Myo9A mutations may underlie a novel form of human autosomal dominant FSGS [49]. Contrary to the aforementioned, RhoA deficiency can also contribute to podocyte cytoskeleton dysfunction in FSGS. The expression and activity of RhoA were significantly diminished in ADR-induced podocyte injury. Mechanistically, inadequate RhoA disturbed the cytoskeleton of podocytes and triggered podocyte apoptosis by inhibiting the Yes-associated protein (YAP)/dendrin signal [50]. In addition, as a crucial regulator of the actin cytoskeleton dynamics, deficient Cdc42 and its downstream effector N-WASP downregulated YAP, thereby facilitating podocyte apoptosis [51].

Rabs, another group of small GTPases, play a predominant role in intracellular vesicle trafficking and are closely associated with cytoskeleton components, functioning in kidney biology [52,53] (Figure 2). The Rab family encompasses over 70 GTPases. Within this extensive family, the expression of Rab3A and its effector Rabphillin-3A (Rph3A) has been identified in podocytes surrounding vesicles within FPs. In patients with FSGS, there is an upregulation of Rab3A-Rph3A expression, potentially leading to cytoskeleton modifications [54,55].

Transient receptor potential channel 6 (TRPC6) is calcium-permeable, and increased calcium influx through TRPC6 is one of the major determinants of podocyte injury in FSGS [56]. Calcium entry via TRPC6 downregulates nephrin and synaptopodin and stimulates the activation of RhoA, leading to actin cytoskeleton rearrangement [57]. Interestingly, feedback from actin cytoskeleton rearrangement modulates calcium entry through TRPC6 [58]. Mutations of the TRPC6 gene are related to autosomal dominant inherited FSGS [59]. TRPC6 knockout podocytes displayed an altered actin cytoskeleton, presenting decreased motility due to a loss of calpain activity. The regulation of calpain activity by TRPC6 was independent of its channel activity, indicating a new mechanism for the pathogenesis of FSGS [60].

Additionally, recent studies have revealed a few molecules that can regulate podocyte cytoskeleton rearrangement in FSGS. A soluble factor of permeability, calcium/calmodulin-dependent serine protein kinase (CASK), is newly found to cause recurrent FSGS after transplantation. Recombinant CASK induces actin cytoskeleton rearrangement in immortalized podocytes, redistributing the junction proteins ZO-1 and synaptopodin [61]. Given the secretion of circulating CASK into the sera of patients with recurrent FSGS, it holds promise as a novel biomarker or therapeutic target for recurrent FSGS, thereby conveniently reflecting renal lesions and potentially reducing the frequency of renal biopsy. However, further comprehensive studies are warranted to validate its feasibility and practicality. Chen and colleagues revealed a FAM40A gene mutation in data from 54 FSGS patients. Mutant FAM40A (p521M > T) altered the mouse podocyte morphology and cytoskeleton with disordered F-actin, loss of FPs, and nephrin, indicating that mutant FAM40A may be associated with familial FSGS [62]. In the adriamycin (ADR) murine model and patients with FSGS, a complement C3 convertase regulator decay-accelerating factor (DAF) was lost, leading to the activation of complement and the formation of C3a. Then C3a/C3aR ligations on podocytes initiated autocrine IL-1β/IL-1R1 signaling, which induced nephrin reduction and cytoskeleton rearrangement, finally leading to glomerulosclerosis [63]. Additionally, the expression of plectin was downregulated in ADR-induced podocyte injury, thereby resulting in cytoskeleton rearrangement and ultimately facilitating podocyte apoptosis through the activation of the integrin α6β4/focal adhesion kinase (FAK)/p38 signaling pathway [64].

Asparagine endopeptidase (AEP) is an endosomal and lysosomal cysteine protease recognized for its proteolytic action to degrade substrates [65]. AEP exhibits high expression in the kidney, and numerous studies have established its indispensability for renal physiology and homeostasis [66,67,68]. Impairment of lysosomal function disrupted the regulation of SD proteins, nephrin, and podocin, leading to FPE [69]. Our recent research revealed that AEP exerted a renoprotective effect in animal models of FSGS by regulating podocyte cytoskeleton rearrangement through its enzymatic activity. In ADR-induced nephropathy, AEP was abundantly expressed in podocytes. AEP cleaved the actin-binding protein transgelin at the N150 site, removing the C terminus of transgelin. The fragment of transgelin was capable of protecting against podocyte injury and restoring F-actin and paxillin, a focal adhesion protein. Collectively, AEP modulates podocyte cytoskeleton rearrangement by exerting a proteolytic role on the actin regulatory protein transgelin. The protective effect of AEP on podocytes provides a potential strategy for the treatment of FSGS [70].

### 4.2. Minimal Change Disease

Minimal change disease (MCD) is one of the primary forms of podocytopathies, and it is the most common NS in children [71].

Alterations in nephrin play a critical role in the pathogenesis of MCD [72]. IQ domain GTPase-activating protein 1 (IQGAP1), a scaffolding protein linking the actin cytoskeleton to the SD, was found to interact with nephrin and regulate the podocyte actin cytoskeleton [73] (Figure 2). IQGAP1 expression and its interaction with nephrin in podocytes were vastly decreased in PAN-induced rats, an animal model of MCD. In podocytes, IQGAP1 knockout significantly reduced the colocalization of IQGAP1 with nephrin and exacerbated the disruption of F-actin. These effects were dramatically restored by IQGAP1 overexpression [74].

In addition, nephrin phosphorylation is known to decrease in patients with MCD [72]. Phosphorylated nephrin was available to bind to the nonreceptor tyrosine kinase c-Abl in the SH2/SH3-dependent form, and c-Abl recruitment depended on the phosphorylation of nephrin. Moreover, nephrin-c-Abl signaling was shown to modulate podocyte cytoskeleton rearrangement. This observation revealed that c-Abl was involved in podocyte cytoskeleton rearrangement, depending on nephrin. It indicated that c-Abl was a novel downstream effector of nephrin in maintaining podocyte cytoskeleton stability. Compared with control patients, nephrin-c-Abl colocalization decreased in the glomeruli of patients with MCD, consistent with previous results in animal models and cultured podocytes [75]. These findings suggested that nephrin-c-Abl signal transduction dynamically regulated the podocyte cytoskeleton and participated in podocyte injury during MCD.

In addition to nephrin, other SD proteins such as podocin, CD2AP, and ACTN4 can be modulated in MCD. Administration of IL-13 substantially downregulated synaptopodin, ACTN4, CD2AP, and ZO-1 in podocytes and thus rearranged the cytoskeleton of podocytes. IL-13 may be involved in the pathogenesis of MCD, and specifically targeting IL-13 could be a promising therapeutic option for MCD [76]. In addition, serum- and glucocorticoid-inducible kinase 3 (SGK3) is a downstream mediator of PI3K, which is essential for maintaining the intact function of podocytes. In PAN-induced podocyte injury in vitro, the activity of SGK3 was decreased, leading to reduced glycogen synthase kinase-3 (GSK3) phosphorylation, and then GSK3 was activated. Consequently, GSK3 activation resulted in decreased expression of podocin, which damaged the podocyte cytoskeleton [77]. Angiopoietin-like protein 4 (ANGPTL4) is a glycoprotein belonging to the angiopoietin-like protein family. ANGPTL4 was markedly increased in the glomerulus in a PAN-induced injury. ANGPTL4 participated in the podocyte cytoskeleton rearrangement by observably downmodulating podocin and ACTN4, thereby promoting FPE and proteinuria [78]. In addition, ANGPTL4 downregulated the expression of synaptopodin, induced podocyte cytoskeleton rearrangement, and promoted podocyte apoptosis in MCD [79].

Recent studies have found the role of autoantibodies in the pathogenesis of MCD. Autoantibodies against nephrin play a vital role in cytoskeleton rearrangement. By evaluating sera from patients with MCD, Watts and colleagues reported that circulating nephrin autoantibodies were present in patients with MCD and were associated with heavy proteinuria. Moreover, the subtle IgG colocalized with nephrin specifically. Based on these observations, the integrity of the SD and the cytoskeleton organization may be disrupted by the binding of nephrin to autoantibodies [80]. In addition, Annexin A2 (ANXA2), a calcium-regulated phospholipid-binding protein, can interact with F-actin and other cytoskeleton-associated proteins. ANXA2 regulates the podocyte cytoskeleton by modulating Rho signaling [81]. The level of ANXA2 autoantibodies was much higher in children with primary MCD, which significantly induced proteinuria. As for the mechanism, the autoantibodies against ANXA2 activated the Cdc42 and Rac1/2/3 Rho pathways by facilitating ANXA2 phosphorylation at Tyr24, thereby resulting in the cytoskeleton rearrangement of podocytes and inducing proteinuria even in primary NS [82]. In brief, ANXA2 was expressed in podocytes and served as the antigen for the autoantibody in patients with primary MCD. It was involved in the pathogenesis of MCD and may be a potential therapeutic target.

### 4.3. Diabetic Kidney Disease

Diabetic kidney disease (DKD) is one of the major complications of diabetes mellitus and is the leading cause of ESRD worldwide [83]. Podocyte cytoskeleton rearrangement is one of the major contributors to the pathogenesis of DKD [84].

The structure of the podocyte cytoskeleton is highly dependent on ATP availability [85]. Podocytes are metabolically active, and their function relies on efficient glucose oxidation for energy production [84]. Chronic hyperglycemia leads to progressive impairment of glucose metabolism in podocytes, greatly hindering mitochondrial ATP production [86]. This failure of ATP synthesis by mitochondria significantly disrupts the balance between cytoskeleton assembly and disassembly, particularly affecting the actin cytoskeleton and MTs, ultimately resulting in the cytoskeleton rearrangement [87,88,89]. Additionally, GTP plays a crucial role in regulating the structure and function of the podocyte cytoskeleton, primarily through its interaction with Rho GTPases [90]. GTP biosynthesis enzymes dynamically regulate the activity of Rho GTPases, which undergo cycling between an inactive GDP-bound state and an active GTP-bound conformation [91]. Moreover, podocytes incubated with HG exhibited a significant increase in Cdc42-GTP level, which correlated with cytoskeleton remodeling [92].

Insulin is a hormone that plays a pivotal role in glucose metabolism and can regulate podocyte cytoskeleton rearrangement, mainly through the modulation of the Rho family (Figure 2). The importance of insulin signaling has been highlighted in the pathogenesis of DKD [93]. Insulin-induced actin cytoskeleton rearrangement occurs via activation of TRPC6 channels, and this activation increases AMPKα phosphorylation and the colocalization of TRPC6 with AMPKα2 subunits. The activation of both TRPC6 and AMPKα2 was necessary to activate Rac1 [94]. Moreover, insulin can regulate Rac1 and RhoA in a protein kinase G type Iα (PKGIα)-dependent manner [95]. PKGI is a type of PKG that belongs to the family of serine/threonine kinases. PKGI is primarily a mediator of vasodilation and was later found to be a major modulator of filtration barrier permeability [96]. The activation of PKGI induced actin cytoskeleton rearrangement and consequently increased podocyte filtration barrier permeability via activation of Rac1 and inhibition of RhoA. This mechanism was disrupted in diabetic circumstances [95]. The appropriate PKGIα-Rac1 interplay is also a major regulator of the PAK/cofilin-dependent signaling pathway in podocytes. The downregulation of PKGIα or Rac1 expression abolished this effect [97]. Accordingly, PKGIα–Rac1–RhoA crosstalk is necessary to properly organize the podocyte cytoskeleton and stabilize glomerular architecture and function (Figure 2).

The role of Rab3A has also been investigated in DKD. Rab3A knockout mice fed with the HG diet exhibited renal lesions similar to those in DKD patients, along with profound alterations in actin cytoskeleton plasticity and proteinuria [98]. Furthermore, the Rab3A/Rab27A system is a core player in podocyte injury and vesicle transport in DKD. Silencing RAB3A/RAB27A in podocytes exposed to HG-induced cytoskeleton disorganization and cellular apoptosis [99]. Considering the crucial involvement of Rabs in vesicle transport and their close association with the podocyte cytoskeleton, detecting the expression of Rabs in urine exosomes holds promise for non-invasive assessment of cytoskeleton disorders and kidney lesions. This approach could potentially reduce the need for repeated kidney biopsy; however, further studies are required to confirm the highly specific association between Rabs and podocyte cytoskeleton disorders as well as renal lesions.

Podocyte cytoskeleton rearrangement is tightly associated with the progress of cellular apoptosis in DKD. The downregulation of RhoA in DKD results in the disruption of the podocyte cytoskeleton, thereby promoting apoptosis by inhibiting YAP. Thus, RhoA/YAP signaling is a promising target for treating DKD [100]. Additionally, tyrosine-protein kinase receptor (TYRO3) expression was largely suppressed in DKD patients and HG-treated podocytes. Knockdown of TYRO3-induced podocyte cytoskeleton rearrangement facilitates cellular apoptosis and exacerbates proteinuria in DKD mice. Mechanistically, TYRO3 protected podocytes from cytoskeleton disturbances and therefore ameliorated apoptosis through the JNK/c-jun-p53 pathway [101]. Under diabetic conditions, ANGPTL4 expression was upregulated, resulting in actin cytoskeleton rearrangement and podocyte apoptosis via activating the integrin-β1/FAK pathway [102]. The expression of brain acid-soluble protein 1 (BASP1), a critical regulator of the actin cytoskeleton, was upregulated in DKD. This upregulation contributed to the rearrangement of the actin cytoskeleton and subsequently promoted podocyte apoptosis by activating the p53 apoptotic pathway through modulation of Wilms’ tumor 1 transcription factor (WT1) [103]. The aforementioned signaling pathways possess the potential to mitigate podocyte cytoskeleton disorders and apoptosis, thereby offering therapeutic prospects for DKD.

A number of molecules have been implicated in the regulation of SD proteins and actin cytoskeleton rearrangement in DKD. The protein tyrosine phosphatase SHP-1 has been shown to modulate SD protein and podocyte cytoskeleton rearrangement. Its expression was increased in the podocytes of diabetic mice and patients with diabetes. Podocyte-specific deletion of SHP-1 prevented FPE and restored the expression of podocin. In terms of the mechanism, SHP-1 reduced SUMO2 post-translational modification of podocin, and inhibition of SHP-1 rescued reduced podocin in diabetic conditions [104]. The expression of MYH9 was significantly decreased in the glomeruli of diabetic patients and mice. In DKD, the reduction of MYH9 induced podocyte injury and actin cytoskeleton rearrangement, which manifested as a decreased expression of nephrin, synaptopodin, ZO-1, and β1 integrin, a focal adhesion protein [105]. In addition, dipeptidyl peptidase-4 (DPP-4) activity was observed in nodular lesions in DKD, which induced degradation of synaptopodin and reduction of RhoA, destroying the podocyte cytoskeleton [106]. Slit–Robo GTP activating protein 2a (SRGAP2a), belonging to the large family of Rho-GTPase, primarily localizes at podocytes and largely colocalizes with synaptopodin. SRGAP2a was downregulated in DKD patients and db/db mice exhibiting disrupted podocyte cytoskeletons, and SRGAP2a knockdown in podocytes rearranged the cytoskeleton by inactivating RhoA/Cdc42 [107]. In addition, by performing RNA sequencing in kidneys isolated from DKD patients, Hu and colleagues reported that the novel lncRNA ENST00000436340 was upregulated in DKD and contributed to cytoskeleton rearrangement with collapsed AFs, upregulation of desmin, and downregulation of ZO-1, ultimately leading to podocyte injury and DKD [108].

Cathepsin L, a lysosomal cysteine protease, was involved in the pathogenesis of DKD [109]. This enzyme cleaves the large GTPase dynamin, CD2AP, and synaptopodin, which are associated with actin cytoskeleton rearrangement of podocytes, FPE, and proteinuria [110,111,112]. Our research group recently discovered that AEP exhibits protective effects against podocyte injury by regulating the cytoskeleton dynamics in DKD. AEP was highly expressed in diabetic kidney podocyte injury and podocytes exposed to hyperglycemia. Mechanistically, AEP directly cleaved the actin-binding protein cofilin-1 at the N138 site. The resulting cofilin-1–1–138 fragment was capable of preventing podocyte cytoskeleton disarrangement and ameliorating podocyte injury in DKD [113].

The discovery of these mechanisms provides a strong rationale for targeting podocyte cytoskeleton rearrangement in DKD.

### 4.4. Membranous Nephropathy

Membranous nephropathy (MN) is a kind of autoimmune glomerular disease typically associated with autoantibodies against the phospholipase A2 receptor (PLA2R) and characterized by apparent thickening of the glomerular capillary walls owing to immune complex deposition [114]. The immune-related mechanisms of MN have been extensively studied, and increasing evidence implicates that podocyte cytoskeleton rearrangement is closely related to the pathogenesis of MN [115].

Ubiquitin C-terminal hydrolase-L1 (UCH-L1), a member of the de-ubiquitinating enzyme families of the ubiquitin–proteasome pathway, was found to express in podocytes. The expression of UCH-L1 was aberrantly increased in the podocytes of patients with MN and the passive Heymann nephritis (PHN) rats, an experimental model of MN [116]. The conspicuous upregulation of UCH-L1 in podocytes resulted in a massive accumulation of p27^Kip1^, which contacted cytoskeleton-associated proteins including RhoA and Rac, therefore regulating the podocyte cytoskeleton [117]. Moreover, upregulation of UCH-L1 could lead to cytoskeleton rearrangement of podocytes with decreased synaptopodin and RhoA [118,119]. However, UCH-L1 damaged the podocyte cytoskeleton and caused MN by suppressing plakoglobin instead of directly acting on synaptopodin and RhoA. Upregulation of UCH-L1 was relevant to a prominent downregulation of plakoglobin, which takes close participation in rearranging podocyte cytoskeleton and maintaining podocyte cytoarchitecture [119]. This finding sheds new insight into the treatment of MN.

In addition, the levels of several molecules are altered in MN, thereby regulating podocyte cytoskeleton rearrangement. The glomerular expression of ANGPTL4 was significantly increased in PHN rats and MN patients. Intriguingly, it was tightly connected with podocyte injury and excessive proteinuria [120]. A subsequent study observed that overexpression of ANGPTL4 resulted in cytoskeleton rearrangement of podocytes, exhibiting decreased expression of synaptopodin. This study provided a potential therapeutic target for MN [79]. In addition, the expression of glomerular Matriptase was dramatically increased in patients with MN. Matriptase directly cleaved podocin, thus disrupting the cytoskeleton of podocytes and the integrity of SD. This finding implies that inhibiting Matriptase could be a potential therapeutic target for podocytopathies [121]. In addition, as mentioned above in MCD, the nephrin-c-Abl colocalization was suppressed in the glomeruli of patients with MN [75].

In 2009, PLA2R was identified as the primary targeted autoantigen in MN. The presence of autoantibodies against PLA2R is highly specific to MN and serves as a valuable biomarker for the diagnosis of MN [114]. Haddad and colleagues recently revealed that in primary MN, antibodies against PLA2R1 combined with podocyte PLA2R1 and thereby activated the complement system, which was promoted by the activation of the lectin pathway in a glycosylation-dependent manner. Subsequently, these effects induced the proteolysis of two podocyte cytoskeleton proteins, synaptopodin and NEPH1, disorganizing the podocyte cytoskeleton [115].

### 4.5. Lupus Nephritis

LN is a kidney disease that primarily injures podocytes. It is characterized by the deposition of autoantibodies, intrinsic and adaptive immune activation, and the production of local mediators associated with renal inflammation and fibrosis [122].

Recently, MiR-26a was identified as a multifunctional mediator of kidney diseases. It dynamically modulates podocyte cytoskeleton organization and is extremely important for podocyte function [123]. MiR-26a expression was significantly decreased in patients with LN as well as in the glomerulus of *B6.MRLc1* mice, a mouse model of autoimmune glomerulonephritis. Decreased MiR-26a was tightly correlated with reduced actin proteins and the IFs protein vimentin, indicating the disruption and rearrangement of the podocyte cytoskeleton [124]. MicroRNAs can be released into urine, and several urinary microRNAs have been identified as potential biomarkers for kidney diseases. Moreover, exosome-derived microRNAs in urine have garnered significant attention [125]. Therefore, further exploration is warranted to assess the utility of exosomal MiR-26a in predicting cytoskeleton disruption and determining therapeutic efficacy, and extensive, robust clinical studies are necessary to validate the clinical value of MiR-26a.

Neutrophils are implicated as a causal regulator in LN and can directly interact with podocytes. Cytokines released by podocytes recruit neutrophils and promote the release of neutrophil granule components, consequently disrupting the actin cytoskeleton. These changes contribute to renal abnormalities and proteinuria in animal models of LN [126]. In addition to MN, the conspicuous upregulation of UCH-L1 was also observed in LN and led to podocyte cytoskeleton rearrangement by decreasing the expression of plakoglobin in podocytes [119].

## 5. Therapeutic Implications

Considering the determinant role of podocytes in glomerular filtration function and the fact that podocyte injury is the hallmark of proteinuric diseases, it is logical to treat podocytes as a specific target for developing novel therapeutic strategies. Additionally, a series of cellular and signaling mechanisms indicate that treatment focusing on cytoskeleton rearrangement may be beneficial for podocytopathies. Currently, existing therapeutic options focus on renal pathophysiology, which has poor specificity, limited therapeutic efficacy, and is often accompanied by significant side effects. However, several studies have confirmed their effects on the podocyte cytoskeleton and the treatment of podocytopathies, which are summarized next to provide potential therapeutic options [127,128] (Table 1).

### 5.1. Glucocorticoids

Glucocorticoids (GCs) are the cornerstone medication for proteinuric diseases, and they exert well-known effects via immunosuppressive and anti-inflammatory responses [129]. An increasing number of studies have indicated that the therapeutic benefits of GCs can be attributed to the direct target of podocytes, attenuating podocyte cytoskeleton rearrangement [130,131].

GCs therapy can regulate the expression or distribution of nephrin and therefore maintain the podocyte actin cytoskeleton and filtration function. Dexamethasone (DEX) promoted process formation and cellular maturation with a striking upregulation of nephrin in human cultured podocytes [132]. Under glucose starvation-induced rapid endoplasmic reticulum (ER) stress, nephrin was not sufficiently glycosylated and remained in the ER. DEX facilitated the synthesis of fully glycosylated nephrin and rescued the disturbed transport of nephrin to the plasma membrane [133]. Prednisone realized antiproteinuric effects through alterations in the expression and distribution of nephrin and other SD proteins such as podocin, CD2AP, and ACTN4 [134]. LN mice with early treatment with GCs exhibited increased expression of nephrin and podocin and preserved histological morphology [135]. Additionally, DEX increased the phosphorylation of nephrin by activating Fyn with the cooperation of Nck [136]. Further studies revealed that GCs also enhanced nephrin phosphorylation by activating serum and glucocorticoid-regulated kinase 1 (SGK1) [137].

In addition to nephrin, GCs can regulate other actin-associated proteins, including TRPC6, ACTN4, Rac1, and synaptopodin. In PAN rats or cultured podocytes, DEX can rescue remarkably reduced processes and maintain SD integrity by blocking TRPC6 signaling and stabilizing TRPC6 expression [138]. DEX can interact with ACTN4 and maintain the balance of ACTN4 expression in cultured murine podocytes; it significantly stabilizes the actin cytoskeleton and prevents ADR-induced actin cytoskeleton rearrangement [139]. GCs were found to decrease the overactivity of Rac1, which was related to proteinuria, and this Rac1 inhibition had therapeutic efficacy and protected podocyte filtration function [140]. In a retrospective study with 17 FSGS patients, reduced synaptopodin expression was connected with an unsatisfactory response to GC treatment [141]. A subsequent study found that GCs increased synaptopodin expression in PAN-injured glomeruli [142]. Additionally, in immortalized mouse podocytes stimulated with vasoactive factors, DEX modulated cyclic guanosine monophosphate (cGMP)-mediated actin rearrangement and podocyte motility [143]. Overexpression of Krüppel-like factor 15 (KLF15), a downstream target crucial for podocyte restoration upon GCs treatment, prevented the disassembly of the actin cytoskeleton and enhanced actin stress fiber formation under cell stress in cultured human podocytes [144,145,146].

### 5.2. Rituximab

Rituximab (RTX), located on the surface of B cells, is a chimeric monoclonal antibody selective against CD20. This drug has been approved for B cell lymphoma and various autoimmune diseases and is also applied for podocytopathies such as FSGS and MCD [147,148].

RTX can directly affect the actin cytoskeleton and prevent the reduced expression of nephrin and podocin [149]. In an in vitro PAN model, treatment with RTX dramatically prevented disruption of the actin cytoskeleton and increased podocyte actin stress fibers, which was related to a substantial enhancement in cell adhesion [150].

In addition to CD20, Perosa and colleagues found a potential reactivity of RTX to sphingomyelin phosphodiesterase acid-like 3b (SMPDL-3b), a putative acid-sphingomyelinase (ASMase) [151]. A later study reported that RTX could directly target podocytes in recurrent FSGS by preserving SMPDL-3b, which modulates actin rearrangement. The total number of SMPDL3b-positive cells was decreased in kidney biopsies from patients with recurrent FSGS after transplantation. In the study, podocytes were exposed to the recurrent FSGS sera in vitro and displayed a striking disruption of the actin cytoskeleton. Surprisingly, pretreatment with RTX reversed these adverse changes by stabilizing SMPDL-3b and ASMase to regulate actin cytoskeleton rearrangement in podocytes [152]. Previously, remission of recurrent FSGS with relapsing proteinuria after transplantation by using RTX has been reported in many instances [153,154,155,156]. As seen in both clinical and experimental contexts, SMPDL-3b is a direct target of RTX and a regulator of the podocyte cytoskeleton. These findings provide a solid basis for RTX to prevent recurrent FSGS after kidney transplantation.

### 5.3. Calcineurin Inhibitor

Calcineurin inhibitors (CNIs), including cyclosporine A (CsA) and tacrolimus (TAC, also known as FK506), are widely used to treat proteinuric kidney disease [157]. CNIs are commonly known to mediate immunosuppressive effects by inhibiting calcineurin and suppressing T cell activation via regulating the nuclear factor of activated T cells (NFAT) signaling [158]. Also, the benefits of CNIs on proteinuria can be attributed to their direct effects on the podocyte actin cytoskeleton beyond their effects on T cells [159].

It has been revealed that in podocytes, activation of calcineurin leads to dephosphorylation of synaptopodin, making it more susceptible to the deleterious degradation by cathepsin L, which influences the actin cytoskeleton and induces FPE and proteinuria [160]. CsA in a non-immune form protected podocytes by blocking the calcineurin-mediated dephosphorylation of synaptopodin and consequently maintaining synaptopodin. This mechanism stabilized the actin cytoskeleton, improved foot process motility, and protected against proteinuria [161]. TAC was shown to have similar effects to CsA [162]. Since synaptopodin is a regulator of RhoA and Cdc42 during cytoskeleton rearrangement, it can be concluded that CsA eventually protects the podocyte actin cytoskeleton by regulating RhoA and Cdc42 signaling [163].

Activation of calcineurin tightly requires TRPC6-mediated calcium influx [164]. Constitutive activation of NFAT in podocytes increased TRPC6 expression and induced severe proteinuria [165]. Downregulating TRPC6 and blocking TRPC6-mediated NFAT activation through calcineurin inhibition contributed to the antiproteinuric effect of CNIs [166]. Another study showed that pretreatment with CNIs could reduce insulin-stimulated TRPC6 expression dependent on the calcineurin pathway in DKD [167]. Calcineurin also directly regulates WAVE1, a key regulator of Arp2/3, playing an essential role in regulating cytoskeleton organization. CsA regulated WAVE1 phosphorylation and significantly decreased WAVE1 expression. By this mechanism, the disorganized actin arrangement was partially rescued both in vivo and in vitro [168]. Recent studies have unveiled novel mechanisms by which CNIs affect the podocyte cytoskeleton in podocytopathies. Shen and colleagues found that overexpression of ANGPTL4 directly induced podocyte cytoskeleton rearrangement and reduced synaptopodin expression. CNIs ameliorated PAN-induced expression of ANGPTL4 by targeting the Calcineurin/NFAT signaling pathway [169]. TAC remarkably reduced ANGPTL4 expression, accompanied by improved FPs and a reduction of proteinuria in PHN rats [120] and ADR rats [170]. Yasuda and colleagues demonstrated that TAC alleviated podocyte damage by increasing the expression of a binding protein of TAC FKBP12, which was expressed along the actin cytoskeleton and maintained process formation in ADR-induced podocyte injury. TAC also enhanced the interaction of FKBP12 with actin-associated proteins 14-3-3β and synaptopodin to maintain F-actin [171]. Moreover, TAC significantly improved the expression of nephrin and podocin in diabetic rats [172] and maintained the microtubule-associated protein 1 light chain 3 alpha (LC3) expression in PAN-induced podocyte injury [173]. In clinical settings, CNIs were effective in FSGS [174] and lupus podocytopathy cases [175].

### 5.4. Abatacept

B7-1 usually expresses on activated B cells and other antigen-presenting cells, and it provides a co-stimulatory signal for T cell activation and survival [176]. Podocytes can be induced to express B7-1 in various animal models, and B7-1 is crucial in the orchestration of the podocyte cytoskeleton and is associated with proteinuria [177]. Abatacept is an inhibitor that targets the co-stimulatory molecule B7-1 [178]. It has been approved for the treatment of rheumatoid arthritis [179] and juvenile idiopathic arthritis [180] and is being explored for treating other autoimmune diseases.

Yu and colleagues proposed abatacept targeting podocytes as a novel therapy for proteinuric diseases. In vitro data showed that B7-1 disrupted β1 integrin activation by specifically interfering with its association with talin, which was blocked by abatacept. Administration of abatacept induces partial or complete remission in patients with B7-1-positive primary FSGS or recurrent FSGS after transplantation [178]. However, the validity was questioned due to the small patient number, concurrent treatment regimens, and so on. In several other cases, patients with FSGS or MCD were either responsive or unresponsive to abatacept [181,182,183,184]. These conflicting findings suggest that targeting B7-1 requires more evidence to prove the efficacy of abatacept in podocytopathies. A pilot study (NCT02592798) to evaluate the safety and efficacy of abatacept (given every 28 days for four cycles) in patients with MCD or FSGS and active proteinuria (implying steroid resistance) has been completed. We excitedly expect the results for publication [185].

In DKD, podocyte B7-1 expression was upregulated. Podocytes cultured in vitro with HG exhibited PI3K-mediated B7-1 upregulation, which was related to the degradation of synaptopodin and the loss of activated α3β1 integrin in deranged AFs. Importantly, abatacept prevented cytoskeleton disruption and adhesion abnormalities. In diabetic mice, in vivo, podocyte B7-1 was induced as well, and abatacept ameliorated proteinuria [186]. Nevertheless, it was reported that B7-1 was not induced in humans and experimental DKD [187,188], and abatacept did not show renoprotective effects in STZ-induced DKD [189]. B7-1 expression was also induced in the podocytes of mice and patients with LN, which was associated with the severity of proteinuria [177,178]. A meta-analysis showed that abatacept was effective for the management of LN patients [190], while two clinical trials failed to prove the beneficial effects of abatacept in patients with LN [191,192].

Above all, B7-1 staining in podocytopathies and the effect of abatacept via targeting podocyte B7-1 remain particularly controversial. Administration of abatacept to treat podocytopathies requires more reliable clinical data.

### 5.5. Others

Dynamin is characterized by its role in endocytosis, which modulates the dynamics of the actin cytoskeleton and vesicle transport [193]. In podocytes, dynamin plays a vital role in maintaining cellular structure and function through its oligomerization to regulate the actin cytoskeleton by stimulating actin polymerization and cross-interacting with AFs [194]. Bis-T-23 promoted actin-dependent dynamin oligomerization and thus facilitated actin stress fiber formation and focal adhesion maturation in a dynamin-dependent manner, resulting in a reduction of proteinuria in cultured mouse podocytes [195,196]. Ono and colleagues recently found that Bis-T-23 prevented proteinuria and podocyte injury in a podocyte-specific O-linked β-N-acetylglucosamine transferase knockout model (Podo-Ogt^y/−^) by the same mechanism [197]. An ex vivo treatment of podocytes that detached from the GBM in two patients with FSGS and MN, respectively, was responsive to Bis-T-23, accompanied by restored cytoskeleton and focal adhesion [198]. These findings implicate the feasibility of modulating dynamin-dependent actin dynamics as a potential therapeutic approach for podocytopathies.

Fasudil is the only RhoA kinase inhibitor approved for clinical use, and it has been reported to exert protective roles in kidney diseases with well-established effectiveness and safety [199]. It attenuated angiotensin II (Ang II)-induced cytoskeleton rearrangement and preserved podocyte mobility by modulating SD proteins, including nephrin and CD2AP [200]. Tian and colleagues recently clarified that in *MRL/lpr* mice (an animal model of LN), Fasudil sustained podocyte actin cytoskeleton by inhibiting CaMK4/RhoA signaling and inducing the release of YAP from the 14-3-3β (a chaperonin protein for YAP) to make up for the podocyte damage [201]. Another Rho kinase inhibitor, Y27632, notably attenuated podocyte actin cytoskeleton rearrangement in Ang II-treated rats [202]. It also stabilized the MTs and promoted FP formation [203].

Moreover, two RAS inhibitors, angiotensin-converting enzyme (ACEI) and Ang II type 1 receptor blocker (ARB), are well-known for hemodynamic effects. They significantly attenuated the reduction of nephrin and proteinuria in PAN nephropathy [204] and in the glomeruli of type 2 diabetic rats [205]. Given that AngII can directly increase the expression of TRPC6, which leads to proteinuria [206], ACEI/ARB may inhibit TRPC6 expression in an AngII-dependent manner and reduce proteinuria [207]. The mTOR-inhibitor everolimus (EV) stabilized the podocyte actin cytoskeleton by preventing PAN-induced inhibition of the RhoA–ROCK–MLC pathway in vitro [208]. It also regulated MT stability through increasing tubulin beta 2B (TUBB2B) and doublecortin domain containing 2 (DCDC2) expression [209].

### 5.6. New Potential Therapeutic Strategies

In recent years, studies have identified several potential therapeutic targets to modulate podocyte cytoskeleton rearrangement in podocytopathies.

The melanocortin-1 receptor (MC1R) is one of the adrenocorticotropic hormone (ACTH) receptors and is the most abundant ACTH receptor in the glomeruli. Glomerular MC1R expression was increased in patients and rat models of MN and FSGS, and MC1R exerted a renoprotective effect in podocytes by stabilizing the actin cytoskeleton. This mechanism was realized by ERK1/2-dependent phosphorylation and inhibition of EGFR signaling, accompanied by the stabilization of synaptopodin and the formation of stress fibers in podocytes [210]. Moreover, MC5R agonists also attenuated proteinuria and prevented PAN-induced disruption of the podocyte actin cytoskeleton with the restoration of synaptopodin [211]. Melanocortin therapy was demonstrated to protect podocytes and reduce proteinuria; it may be promising to be a beneficial agent to treat patients with podocytopathies [212,213].

In addition, the angiotensin II type-2 receptor (AT2R) was shown to protect against podocyte injury in FSGS. AT2R agonist compound 21 (C21) stabilized the podocyte actin cytoskeleton and restored the expression of synaptopodin by inhibiting cathepsin L, thus maintaining the integrity of podocyte structure and filtration function [214]. Bardoxolone-methyl (CDDO-Me) is a synthetic triterpenoid known for its anti-inflammatory, antioxidant, and anti-apoptosis effects. In ADR-induced nephropathy, CDDO-Me can modulate the cytoskeleton rearrangement of podocytes. By activating the NRF2 pathway, CDDO-Me enhanced SRGAP2a expression to stabilize the podocyte cytoskeleton [215].

A DPP-4 inhibitor is a kind of drug used to treat type 2 diabetes mellitus, and recently it was found to protect podocytes independent of its hypoglycemic role. DPP-4 activity was detected in the podocytes of DKD and can be considered a biomarker of kidney disease. Treatment with the DPP-4 inhibitor saxagliptin dramatically reduced proteinuria and stabilized the expression of nephrin, synaptopodin, and RhoA [106]. DPP-4 inhibition prevented the reduced expression of podocin as well [216]. Metformin is another drug widely used to treat type 2 diabetes. Recently, it was observed to modulate cytoskeleton dynamics by increasing the expression of nephrin and stabilizing the expression of the TRPC6 channel dependent on AMPKα1 activation and regulation of Rho-family small GTPase activity in diabetic conditions. Eventually, metformin treatment reduced glomerular filtration barrier permeability and proteinuria [217]. These findings may broaden the use of these anti-diabetic drugs and provide more options for the treatment of DKD.

Klotho, primarily identified as the anti-aging gene, was recently demonstrated to function in kidney diseases. Klotho administration attenuated actin cytoskeleton disruption and improved podocyte filtration function. It restored the expression of nephrin and ZO-1 and attenuated the increased TRPC6 expression [218]. A subsequent study found that klotho protected against podocyte cytoskeleton disruption and podocyte injury in DKD by significantly inhibiting the expression of TRPC6 [219]. These studies provide evidence that klotho is a potential therapeutic strategy for treating DKD by preserving the podocyte cytoskeleton.

Moreover, catalpol, as an ingredient from the traditional Chinese medicinal herb Rehmannia glutinosa, could ameliorate podocyte injury in DKD by stabilizing the podocyte cytoskeleton. Treatment with catalpol markedly improved the decrease in nephrin and synaptopodin expression. It also rescued disrupted podocyte cytoskeletons by inhibiting RhoA and Cdc42 activation [220]. A compound phloretin from a natural plant was capable of restoring the expression of nephrin and podocin in DKD, thus protecting the podocyte cytoskeleton and the integrity of SD [221]. In PAN-induced podocyte injury, the expression of ACTN4 and CD2AP was significantly reduced, and Shenqi granule was able to upregulate the expression of ACTN4 and CD2AP [222].

In addition to serving as a diagnostic tool for cytoskeleton disruption and kidney diseases, recent studies have suggested that exosomes can also be utilized as a therapeutic means. Wang and colleagues hypothesized that intramuscular administration of miR-29 encapsulated in exosomes could counteract renal fibrosis induced by unilateral ureteral obstruction (UUO) [125]. Therefore, supplementation of exosomes carrying proteins or microRNAs related to podocyte cytoskeleton rearrangement is anticipated to represent a novel strategy for alleviating cytoskeleton disorganization and renal lesions. Nevertheless, it is worth noting that the isolation, purification, and storage of exosomes remain formidable challenges. The specificity, efficacy, and potential side effects of exosomes still require large clinical studies for validation [223,224].

**Table 1:** Agents that can modulate podocyte cytoskeleton rearrangement.

Agents	Medicine	Mechanisms/Pathways	References
GCs	DEX	Upregulating nephrin and increasing nephrin phosphorylation	[132,136,137]
		Facilitating nephrin synthesis and transporting nephrin to the plasma membrane	[133]
		Blocking TRPC6 signaling and stabilizing TRPC6 expression	[138]
		Stabilizing ACTN4 expression	[139]
		Inhibiting Rac1 overactivity	[140]
		Increasing synaptopodin expression	[142]
/	RTX	Preventing reduced expression of nephrin and podocin	[149]
		Stabilizing SMPDL-3b	[152]
CNIs	CsA	Maintaining synaptopodin by blocking the calcineurin-mediated dephosphorylation of synaptopodin	[161]
		Downregulating TRPC6 expression	[166]
		Decreasing WAVE1 expression	[168]
	TAC	Reducing Angptl4 expression	[120,170]
		Increasing FKBP12 expression and enhancing the interaction of FKBP12 with 14-3-3β and synaptopodin	[171]
		Increasing nephrin and podocin expression and maintaining MAP1 LC3	[172,173]
/	Abatacept	Stabilizing β1 integrin activation and its association with talin	[178]
/	Bis-T-23	Promoting actin-dependent dynamin oligomerization	[195,196,197]
Rho kinase inhibitor	Fasudi	Modulating nephrin and CD2AP	[200]
		Inhibiting CaMK4/RhoA signaling and inducing the release of YAP	[201]
	Y27632	Stabilizing actin cytoskeleton and MTs	[202,203]
RAS inhibitor	ACEI/ARB	Stabilizing nephrin expression	[204,205]
		Inhibiting TRPC6 expression	[207]
mTOR-inhibitor	EV	Preventing inhibition of the RhoA–ROCK–MLC pathway Stabilizing MTs	[208,209]

## 6. Conclusions and Perspectives

In the last few years, the coordination of the podocyte cytoskeleton to sustain intact podocyte structure and function has been strongly emphasized. A large number of mechanisms contributing to cytoskeleton rearrangement in podocytopathies emerged. Nevertheless, it is worth noting that the pathophysiology of podocytes is an evolving scientific issue, so there is an urgent need to constantly explore more valuable mechanisms and improve our understanding of the pathogenesis. Although several drugs have been identified to act on the podocyte cytoskeleton in podocytopathies, the specific mechanisms and the clinical efficacy remain controversial and ambiguous. Therefore, a more in-depth perspective on elaborate signaling cascades or molecules governing podocyte cytoskeleton rearrangement will pave novel avenues for specifically and precisely treating podocytopathies and podocyte-centric diseases.

## Data Availability

Not applicable.
